# Metal Nanoparticles Immobilized on Molecularly Modified Surfaces: Versatile Catalytic Systems for Controlled Hydrogenation and Hydrogenolysis

**DOI:** 10.1021/acs.accounts.1c00013

**Published:** 2021-04-06

**Authors:** Alexis Bordet, Walter Leitner

**Affiliations:** † Max Planck Institute for Chemical Energy Conversion, Stiftstraße 34-36, 45470 Mülheim an der Ruhr, Germany; ‡ Institut für Technische und Makromolekulare Chemie, RWTH Aachen University, Worringerweg 2, 52074 Aachen, Germany

## Abstract

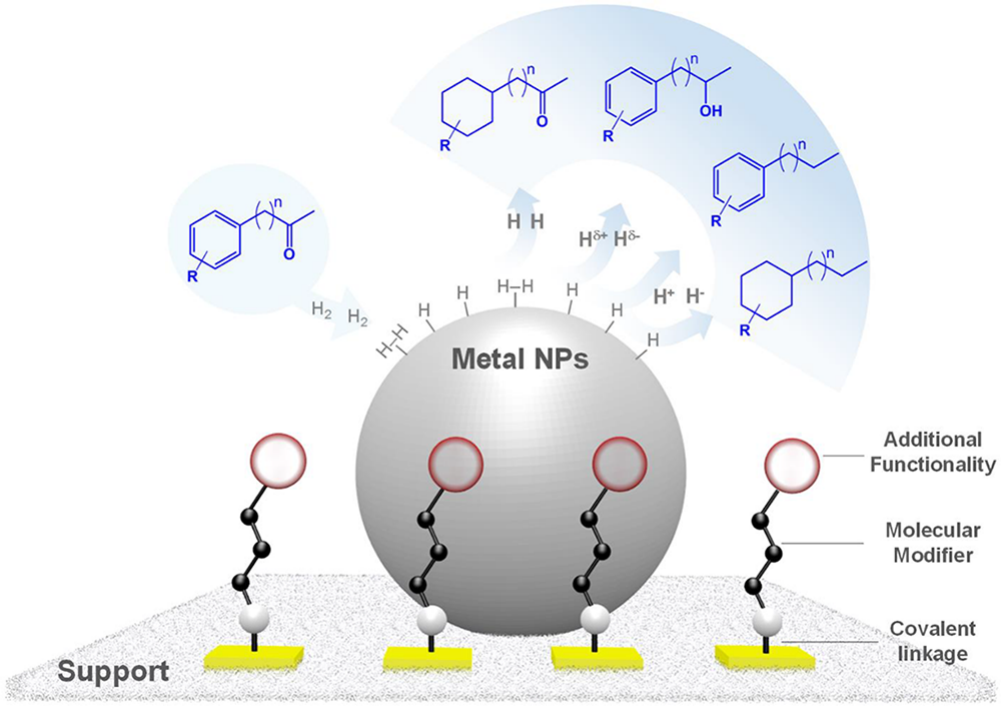

The synthesis and use of supported metal nanoparticle catalysts have a long-standing tradition in catalysis, typically associated with the field of heterogeneous catalysis. More recently, the development and understanding of catalytic systems composed of metal nanoparticles (NPs) that are synthesized from organometallic precursors on molecularly modified surfaces (MMSs) have opened a conceptually new approach to the design of multifunctional catalysts (NPs@MMS). These complex yet fascinating materials bridge molecular (“homogeneous”) and material (“heterogeneous”) approaches to catalysis and provide access to catalytic systems with tailor-made reactivity through judicious combinations of supports, molecular modifiers, and nanoparticle precursors. A particularly promising field of application is the controlled activation and transfer of dihydrogen, enabling highly selective hydrogenation and hydrogenolysis reactions as relevant for the conversion of biogenic feedstocks and platform chemicals as well as for novel synthetic pathways to fine chemicals and even pharmaceuticals. Consequently, the topic offers an emerging field for interdisciplinary research activities involving organometallic chemists, material scientists, synthetic organic chemists, and catalysis experts.

This Account will provide a brief overview of the historical background and cover examples from the most recent developments in the field. A coherent account on the methodological and experimental basis will be given from the long-standing experience in our laboratories. MMSs are widely accessible via chemisorption and physisorption methods for the generation of stable molecular environments on solid surfaces, whereby a special emphasis is given here to ionic liquid-type molecules as modifiers (supported ionic liquid phases, SILPs) and silica as support material. Metal nanoparticles are synthesized following an organometallic approach, allowing the controlled formation of small and uniformly dispersed monometallic or multimetallic NPs in defined composition. A combination of techniques from molecular and material characterization provides a detailed insight into the structure of the resulting materials across various scales (electron microscopy, solid-state NMR, XPS, XAS, etc.).

The molecular functionalities grafted on the silica surface have a pronounced influence on the formation, stabilization, and reactivity of the NPs. The complementary and synergistic fine-tuning of the metal and its molecular environment in NPs@MMSs allow in particular the control of the activation of hydrogen and its transfer to substrates. Monometallic (Ru, Rh, Pd) monofunctional NPs@MMSs possess excellent activities for the hydrogenation of alkenes, alkynes, and arenes for which a nonpolarized (homolytic) activation of H_2_ is predominant. The incorporation of 3d metals in noble metal NPs to give bimetallic (FeRu, CoRh, etc.) monofunctional NPs@MMSs favors a more polarized H_2_ activation and thus its transfer to the C=O bond, while at the same time preventing the arrangement of noble metal atoms necessary for ring hydrogenation. The incorporation of reactive functionalities, such as, for example, a −SO_3_H moiety on NPs@MMSs, results in bifunctional catalysts enabling the heterolytic cleavage corresponding to a formal H^–^/H^+^ transfer. Consequently, such catalysts possess excellent deoxygenation activity with strong synergistic effects arising from an intimate contact between the nanoparticles and the molecular functionality.

While many more efforts are still required to explore, control, and understand the chemistry of NPs@MMS catalysts fully, the currently available examples already highlight the large potential of this approach for the rational design of multifunctional catalytic systems.

## Key References

- Bordet, A.; Moos, G.; Welsh, C.; License, P.; Luska, K. L.; Leitner, W. Molecular Control of the Catalytic Properties of Rhodium Nanoparticles in Supported Ionic Liquid Phase (SILP) Systems.ACS Catal.2020, 10, 13904–139123334399810.1021/acscatal.0c03559PMC7737233.^1^*Influence of the ionic liquid structure in SILPs on the formation of Rh nanoparticles and their catalytic properties for the hydrogenation of biomass-derived furfuralacetone. Comparison with nonmolecularly modified surfaces*.
- Rengshausen, S.; Van Stappen, C.; Levin, N.; Tricard, S.; Luska, K. L.; DeBeer, S.; Chaudret, B.; Bordet, A.; Leitner, W. Organometallic Synthesis of Bimetallic Cobalt–Rhodium Nanoparticles in Supported Ionic Liquid Phases (Co_*x*_Rh_100–*x*_@SILP) as Catalysts for the Selective Hydrogenation of Multifunctional Aromatic Substrates.Small2021, 17, 2006683.10.1002/smll.20200668333346403(2)*Presentation of a versatile organometallic approach to prepare bimetallic CoRh NPs of tunable composition in SILPs. Crucial role of the SILP in the formation of bimetallic NPs and very significant influence of the Co/Rh ratio on the hydrogenation performances of Co*_*x*_*Rh*_*100–x*_*@SILP materials*.
- El Sayed, S.; Bordet, A.; Weidenthaler, C.; Hetaba, W.; Luska, K. L.; Leitner, W. Selective Hydrogenation of Benzofurans Using Ruthenium Nanoparticles in Lewis Acid-Modified Ruthenium-Supported Ionic Liquid Phases.ACS Catal.2020, 10, 2124–2130.(3)*Preparation of Ru nanoparticles on a Lewis acidic SILP. Resulting bifunctional catalyst highly selective for the partial hydrogenation of benzofuran derivatives*.
- Offner-Marko, L.; Bordet, A.; Moos, G.; Tricard, S.; Rengshausen, S.; Chaudret, B.; Luska, K. L.; Leitner, W. Bimetallic Nanoparticles in Supported Ionic Liquid Phases as Multifunctional Catalysts for the Selective Hydrodeoxygenation of Aromatic Substrates.Angew. Chem., Int. Ed.2018, 57, 12721–12726.10.1002/anie.201806638PMC617531930176102(4)*Description of a versatile method to introduce additional functionalities in NPs@SILP materials without affecting the NPs synthesis. Fe*_25_*Ru*_75_*@SILP+IL-SO*_3_*H bimetallic bifunctional catalytic system able to selectively hydrodeoxygenate aromatic ketones while conserving the aromaticity*.

## Introduction

With the depletion of fossil resources and the rise of alternative renewable energy sources and chemical feedstock, the permanent evolution and adaptation of catalysts are of critical importance.^5−7^ Following the recognition of the importance of well-defined small metal nanoparticles in heterogeneous catalysis, the field literally exploded, generating a plethora of nanoparticle-based catalytic systems applied to myriad transformations.^8,9^ In particular, catalysts comprising metal nanoparticles immobilized on various support materials were found to be extremely versatile.^9,10^ While the supports were initially used primarily to stabilize the nanoparticles and to allow for easy recycling, it became obvious that they are rarely innocent and can influence the nanoparticles stability and reactivity through various types of interactions. The support thus became increasingly recognized as an additional crucial optimization parameter of the catalytic systems.^9,11,12^ More recently, the variation and complexity of support materials for catalytically active nanoparticles expanded through the development of surface functionalization techniques. This opened the way for the production of hybrid organic–inorganic materials consisting of molecularly modified surfaces (MMSs).^7,13−19^ In particular, the molecular imprinting of solid supports,^13,14^ the development of SILP (supported ionic liquid phase) and SCILL (solid catalyst with an ionic liquid layer) catalysts,^15,16^ and the immobilization of polymeric structures^17,18^ received great attention, leading to novel design options for solid catalyst materials.

Metal nanoparticles immobilized on MMSs (NPs@MMSs) are complex multicomponent systems but possess several attractive features compared to classical NPs@support catalysts comprising solely inorganic oxides as supports. Potential advantageous properties have been reported to include *inter alia* enhanced NPs stability,^1,11,20,21^ control over NP growth,^1^ tunable wettability,^15,18^ enhanced mass transport,^15,22^ and the introduction of additional functionalities.^22,23^ Most importantly, their versatile and modular synthetic accessibility from defined molecular precursors and materials allows countless variations in the combination of supports, molecular modifiers, and nanoparticles and is thus highly suitable for the development of multifunctional catalytic systems with tailor-made reactivity.^10,22^

Despite a number of pioneering contributions and a highly dynamic recent development, it is fair to say that the potential of NPs@MMS catalytic systems has only been tapped upon until now. We will describe in this Account principal methods to synthesize and characterize NPs@MMS materials and highlight their promising properties in the controlled activation of molecular hydrogen for its selective transfer to various unsaturated functional groups. A particular focus will be placed on the organometallic synthesis of metal nanoparticles on MMSs produced through the covalent attachment of organic compounds on porous solids. Some digressions toward materials obtained through physisorption techniques and alternative NP syntheses will be made when necessary. While a broad range of molecular modifiers can be envisaged in this approach, we will lay special emphasis on ionic liquid (IL)-type structures *pars pro toto* for the general strategy.

## Discussion

### Synthesis and Characterization of NPs@MMS Catalysts

#### Synthesis and Characterization of MMS

The molecular modification of porous solids (supports) has been accomplished through the development of physisorption and chemisorption techniques. When considering physisorption, the organic molecules are immobilized on the support via weak interactions such as dispersion forces, hydrogen bonds, or stronger electrostatic interactions. In contrast, chemisorption implies a covalent attachment of the organic molecules at the surface of the support. While both approaches present advantages and drawbacks, chemisorption is generally preferred when possible, since the resulting materials are typically more stable, well-defined, and less prone to leaching.^13,16,17,22^

**Figure 1:**
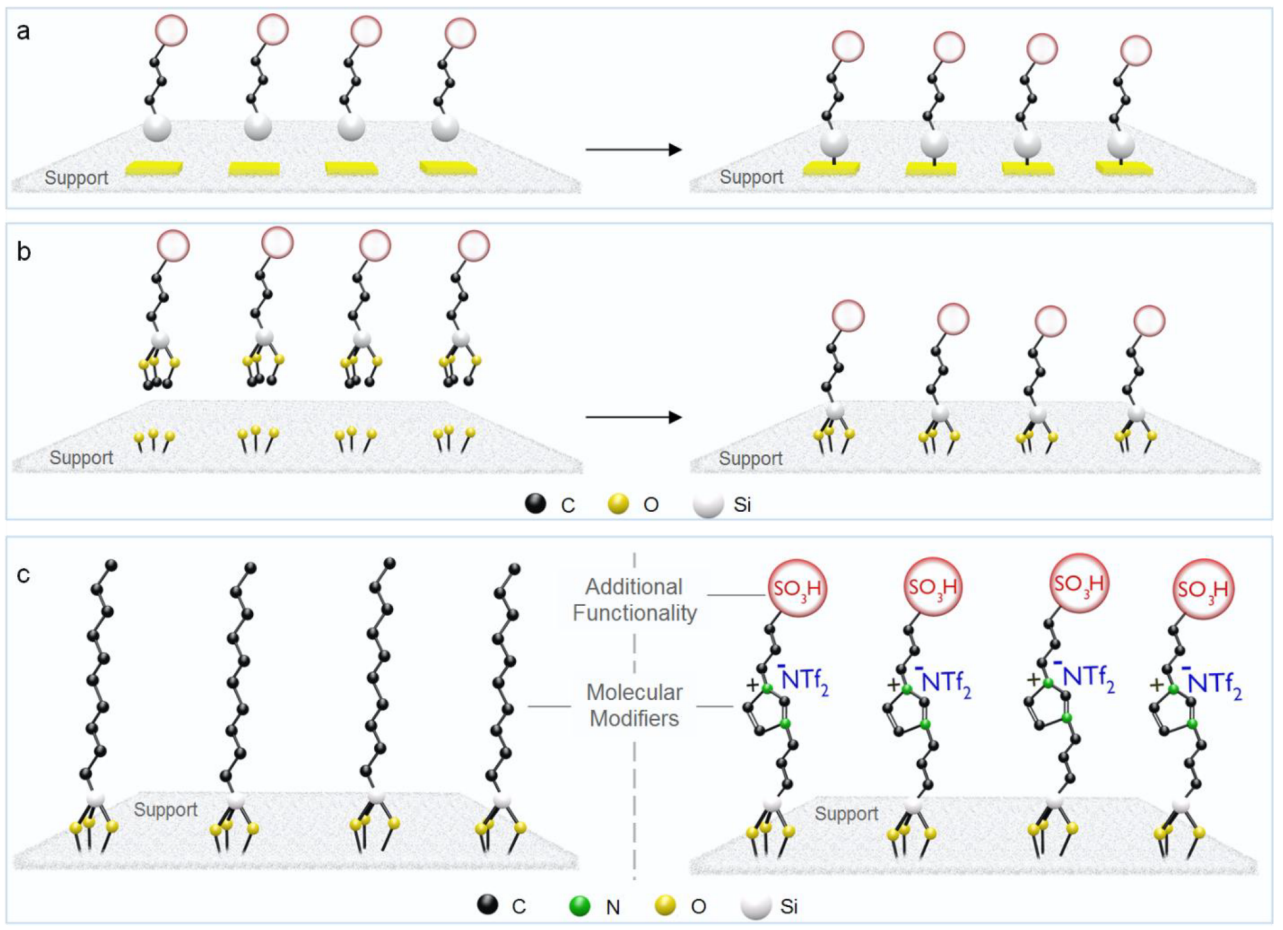
(a) Illustration of the *grafted to* approach for chemisorption; (b) silanization; (c) examples of molecular modifiers chemisorbed through silanization (left: organosilane; right: sulfonic acid functionalized imidazolium-based ionic liquid).

Many techniques have been developed in the past decade to achieve the chemisorption of various organic modifiers on solid supports (e.g., metal oxides, carbon-based supports, etc.).^13,16,17,19,22,24^ When considering the chemisorption of polymers on solid surfaces, the *grafted from* method is popular. In this case, the polymer chains grow *in situ* from an initiator, which has been previously anchored to the support surface.^17,18,24^ Another widely used and highly versatile approach is the so-called *grafted to* method, which involves the reaction of a molecule possessing a reactive end at the surface of a support to form a covalent bond (Figure 1).^17^ A typical example of such grafting is the silanization of oxide supports (e.g., SiO_2_, Al_2_O_3_, TiO_2_, etc.) with alkoxysilane-functionalized molecules. This method produces particularly robust MMSs and has been used to functionalize the surface of solid supports with a wide range of organic compounds possessing various functionalities including small organic molecules,^13,14,19,20,25^ ionic liquids,^1−4,15,16,21−23^ or polymers.^17−19,24^ Such molecular modifiers can be relatively complex and expensive to produce, hindering their direct use in large volumes as solvents for industrial processes. In contrast, the preparation of MMSs requires only small quantities of molecular modifiers and generates materials that are stable and easy to reuse, thus reducing the cost factors and improving their effectivity significantly. Most of the materials discussed in this Account have been obtained by variations of this general methodology.

The influence of the chemisorption of organic compounds on the textural, morphological, and structural properties of the solid supports can be characterized by N_2_ adsorption experiments, electron microscopy, and diffraction techniques. In particular, N_2_ adsorption experiments can give information on the modification of the surface area, pore diameter, and pore volume caused by the functionalization. Scanning electron microscopy (SEM) and transmission electron microscopy (TEM) give an overview of the materials’ morphology and show evidence of the major changes. In the case of crystalline supports, X-ray diffraction (XRD) is the technique of choice to investigate potential structural changes.

**Figure 2:**
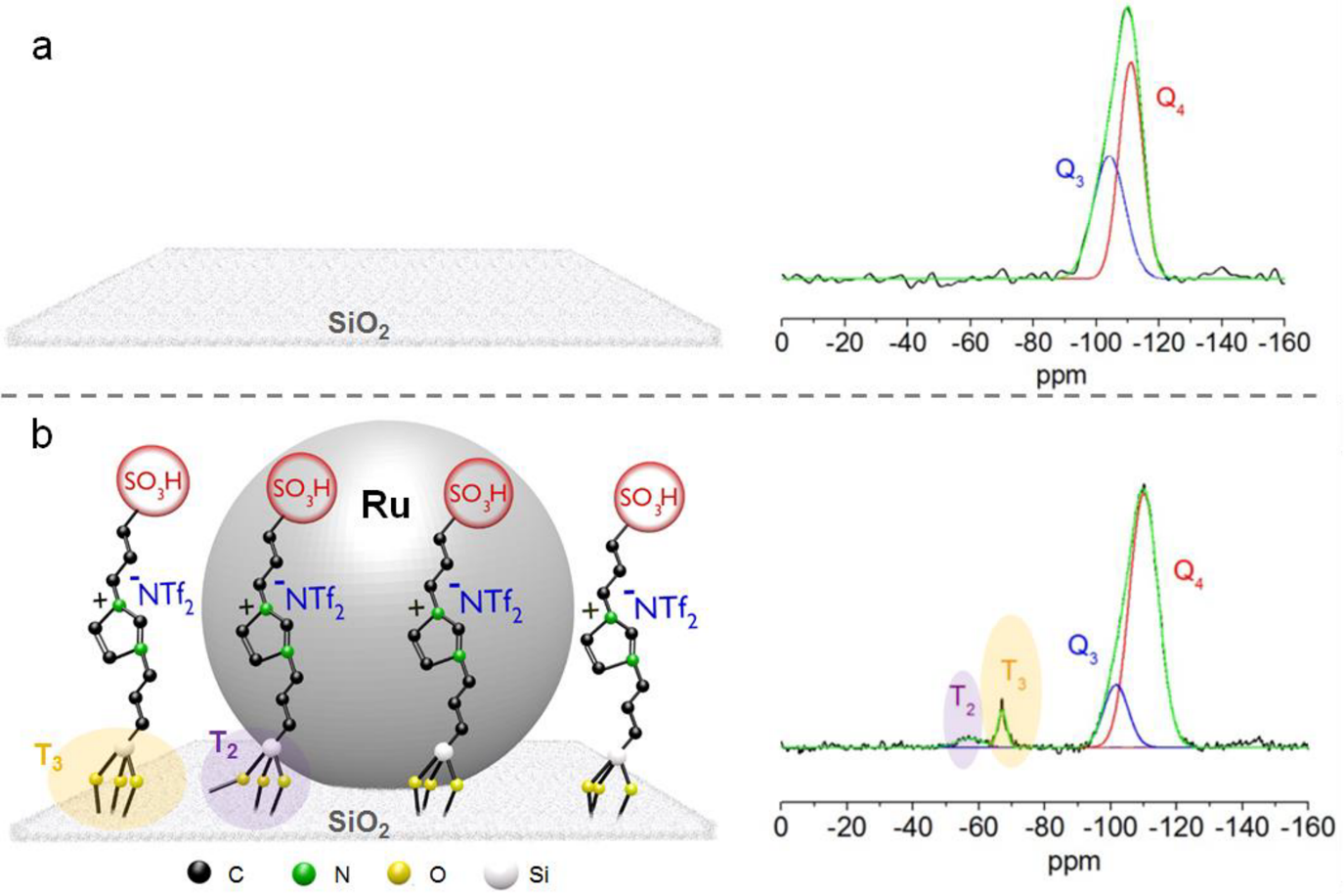
Solid-state ^29^Si CP-MAS NMR of (a) naked SiO_2_ support; (b) SiO_2_ functionalized with an imidazolium-based ionic liquid and bearing Ru NPs.

Solid-state NMR can be used to confirm the covalent attachment of organic compounds to the support. In particular, the connectivity of the siloxy groups through one, two, or three oxygen atoms is readily distinguished by ^29^Si NMR. In Figure 2, this is exemplified for an imidazolium-based IL-type modifier chemisorbed on silica through silanization. The characterization of the resulting SILP material by solid-state ^29^Si CP-MAS NMR showed the presence of trifunctionalized signals (T_3_: R-Si(OSi)_3_; T_2_: R-Si(OSi)_2_(OR′)) corresponding to Si atoms of the ionic liquid bound to the SiO_2_ surface, thus evidencing the covalent attachment of the IL on the silica support (Figure 2).^23^

Other NMR active nuclei such as naturally abundant ^31^P and ^19^F or labeled ^15^N or ^13^C in the molecular modifier may also be used for diagnostics. IR spectroscopy (e.g., transmission, ATR, DRIFTS, etc.) can be used to examine bands that are characteristic of the grafted molecules, thus confirming both their presence and their preserved structure/functionality.^1,23^ In addition, elemental mapping using SEM with energy dispersive X-ray spectroscopy (SEM-EDX) provides valuable information concerning the homogeneity of the surface coverage.

#### Synthesis and Characterization of NPs@MMS

The development of physical and chemical methods allowing the preparation of metal nanoparticles has received broad attention in the past decades. In the context of the present Account, the organometallic approach (*bottom-up chemical method*) has been demonstrated to be particularly powerful to produce size- and shape-controlled nanoparticles possessing well-defined physicochemical properties.^26−28^ This approach, developed in particular by Chaudret and co-workers, involves the thermal decomposition of high energy organometallic complexes or hydrogenation of labile hydrocarbon ligands under relatively mild conditions, avoiding the use of potential sources of pollution arising from the precursor or from the reducing agent.^26,28^ The nanoparticle formation can be achieved directly from the solution or after impregnation of the precursor complex on the MMSs, producing NPs@MMS materials that can be used directly in catalysis without the need for additional prereduction treatment. The simplicity and versatility of this method combined with its capacity to produce various types of metal nanoparticles with practically clean surfaces makes this approach particularly useful for the generation of NPs on the MMSs.

**Figure 3:**
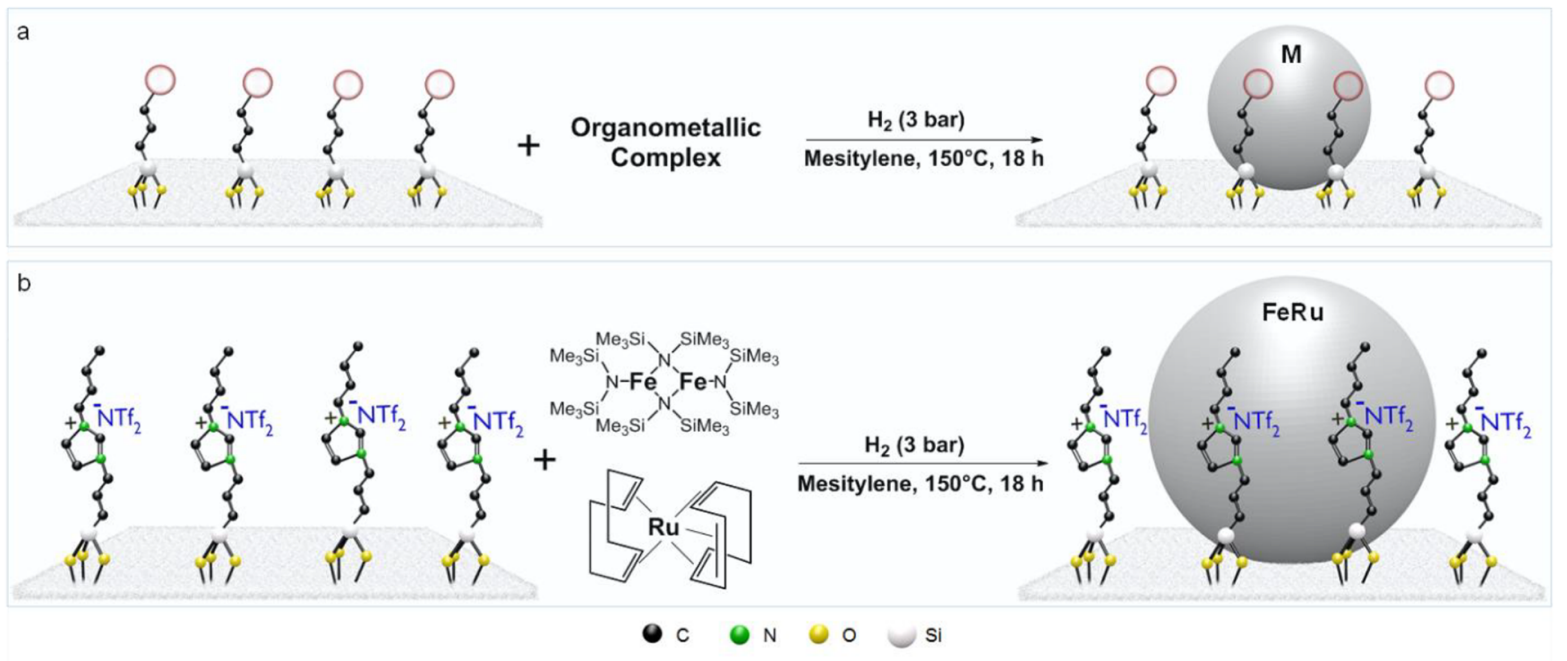
Organometallic synthesis of metal nanoparticles on MMS: (a) general approach; (b) example of bimetallic FeRu NPs immobilized on an imidazolium-based SILP.^31^

In our experience, the organometallic approach has proven to be highly versatile for the generation of well-defined NPs in the 1–5 nm range from various metals and mixtures of metals, including, but not limited to, Fe, Co, Ni, Ru, Rh, Fe_*x*_Ru_100–*x*_, Co_*x*_Ru_100–*x*_, Ni_*x*_Ru_100–*x*_, or Co_*x*_Rh_100–*x*_ (Figure 3a). The preparation of NPs@MMS is routinely accomplished on a 1–10 g scale in our laboratories using standard equipment. As demonstrated for related SILP and SCILL catalysts, their preparation can also be scaled-up to a pilot or even commercial scale when suitable adjustments and optimizations are made. The molecular modification of the support was found to have a strong influence on the growth and stability of the NPs as well as on their reactivity.^1−3,32^ Interestingly, while bimetallic catalysts have increasing importance in catalysis,^29,30^ this method is highly suited to the synthesis of bimetallic M_1_M_2_@MMS catalysts, where the M_1_/M_2_ metal ratio can be finely tuned by changing the relative amount of organometallic complexes introduced (Figure 3b).^2,4,31^ For a consistent nomenclature, we propose the definition of the M_1_ > M_2_ order to follow the 3d > 4d > 5d metal order as well as the atomic number order (e.g., Fe_*x*_Ru_100–*x*_@MMS and not Ru_*x*_Fe_100–*x*_@MMS; Pd_*x*_Ag_100–*x*_@MMS and not Ag_*x*_Pd_100–*x*_@MMS).

**Figure 4:**
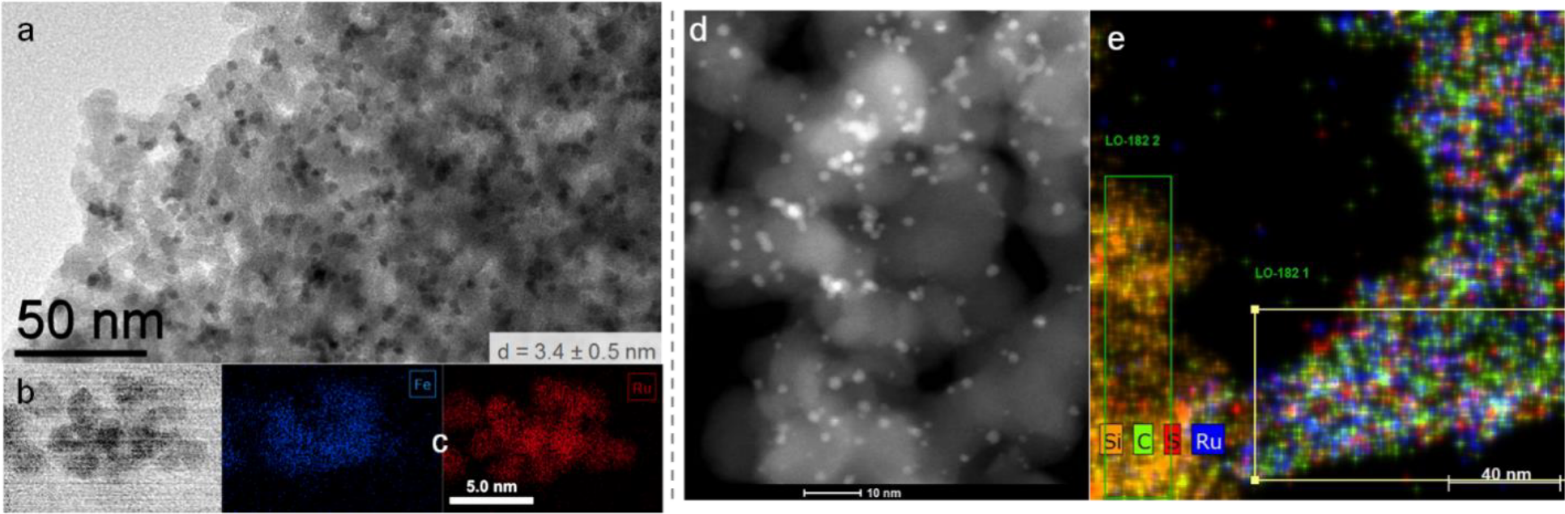
Characterization of NPs@MMS materials using electron microscopy. Left: Fe_25_Ru_75_@SILP catalyst^31^ characterized by (a) TEM; (b) STEM; (c) STEM-HAADF-EDX with elemental mapping. Right: Ru@SILP-SO_3_H catalyst^23^ characterized by (d) STEM-HAADF; (e) STEM-HAADF-EDX with elemental mapping.

X-ray spectroscopy techniques can be used to determine the oxidation state of the metals (XPS and XANES),^1−3,31^ the alloying extent in the case of bimetallics (EXAFS)^2,31^ and to study the electronic interactions between the NPs and the MMS (XPS).^1,3^ SEM, TEM, and scanning transmission electron microscopy with energy dispersive X-ray spectroscopy (STEM-EDX) provide information about the size of the NPs, their dispersion on the support, their composition, their interaction with the molecular modifier, and their bimetallic nature when applicable (see examples in Figure 4).^1−4,31^ For example, Figure 4e shows a STEM-HAADF-EDX (HAADF, high angle annular dark field) image of a catalyst composed of Ru NPs immobilized on a sulfonic acid functionalized imidazolium-based SILP (Ru@SILP-SO_3_H). The elemental mapping evidences that the Ru NPs are concentrated in the zones containing the ionic liquid layer, providing direct evidence for the stabilizing effect of the IL environment and the close contact of the metal NP and the molecular functionality.

A summary of the key information required to characterize MMS and NPs@MMS as well as the associated techniques is provided in Table 1.

**Table 1:**
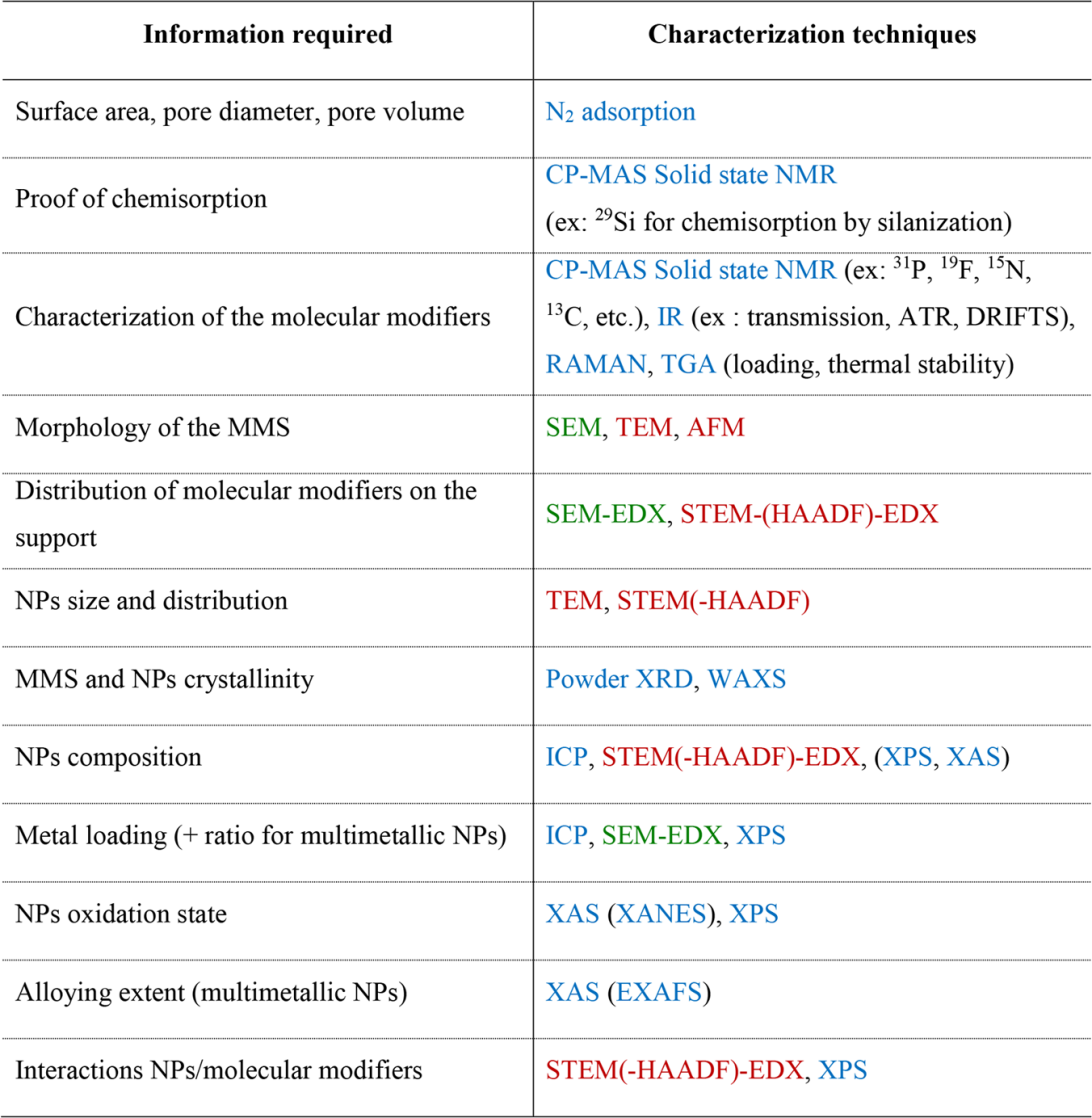
Selection of the Characterization Techniques Suitable for the Study of MMS/NPs@MMS Materials^a^

a Characterization techniques provide information on the global (bulk; blue text), intermediate (ca. 500–0.1 μm; green text), and local (ca. 500–1 nm; red text) scale.

After catalysis, the same techniques can be used to investigate the potential changes in NPs@MMS materials including leaching of the molecular modifier, leaching of the metal, aggregation, and growth of the metal nanoparticles.

### Reactivity: Activation of Dihydrogen for the Conversion of Unsaturated Functional Groups in Aromatic Substrates

**Figure 5:**
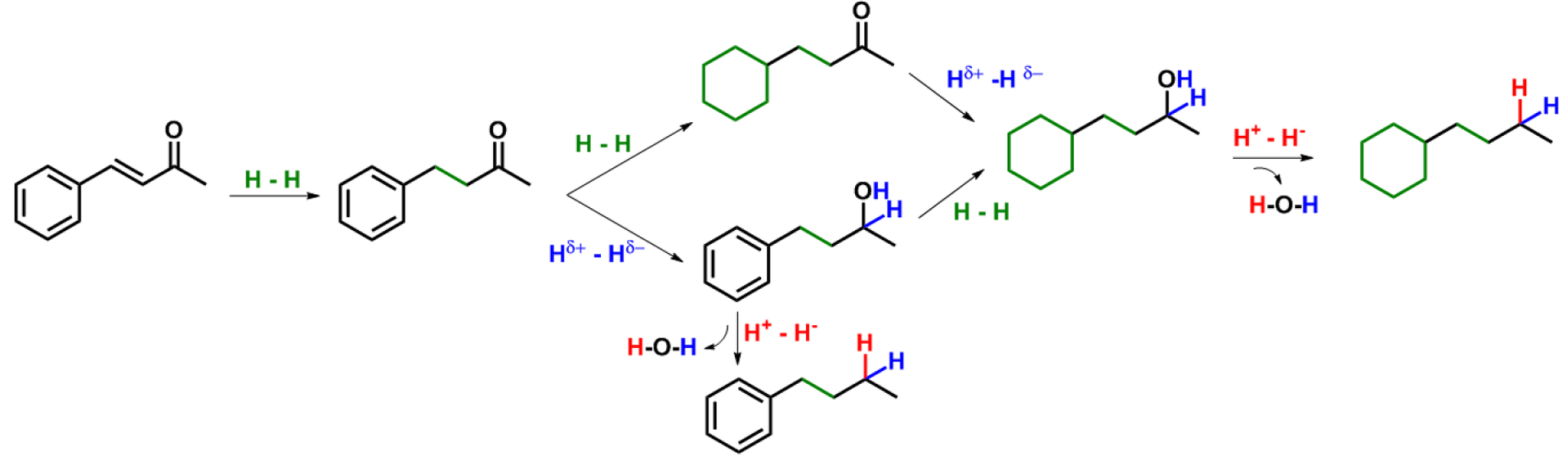
Reaction pathway for the hydrogenation and hydrodeoxygenation of benzylideneacetone, evidencing the H_2_ activation requirements for each transformation.

We will describe in this part recent advances in the use of NPs@MMS catalysts for the activation of molecular hydrogen and the selective hydrogenation and hydrogenolysis of unsaturated functional groups in various aromatic substrates. The concept takes advantage of the modularity of NPs@MMS catalytic systems to control the elementary processes of how molecular hydrogen is activated and transferred to substrates. The requirements to hydrogenate alkenes, aromatic rings, or ketones or to hydrogenolytically deoxygenate alcohols are different especially in terms of hydrogen polarization,^10^ as highlighted in Figure 5 using benzylideneacetone as a prototypical example for substrates with several potentially reducible moieties. Typically, the hydrogenation of alkenes and aromatics can be achieved with H_2_ activated through homolytic dissociation (H–H), while the hydrogenation of ketones requires more polarized hydrogen activation (H^δ+^–H^δ−^) resulting in formal hydride and proton delivery.^10^ Going one step further, the deoxygenation of alcohols involves dehydration via carbocation intermediates or transition states, and the presence of acidic protons from the heterolytic cleavage of hydrogen (H^+^–H^–^) is expected to facilitate theses elementary steps.

In the following sections, we present selected recent efforts from our laboratories and other teams to exploit the modularity of NPs@MMS catalytic systems in this context. The discussion will be organized according to the metal component (mono- vs bimetallic) and the functionality of the molecular modifier (mono- vs multifunctional).

#### Monometallic Monofunctional NPs@MMS Catalysts

**Figure 6:**
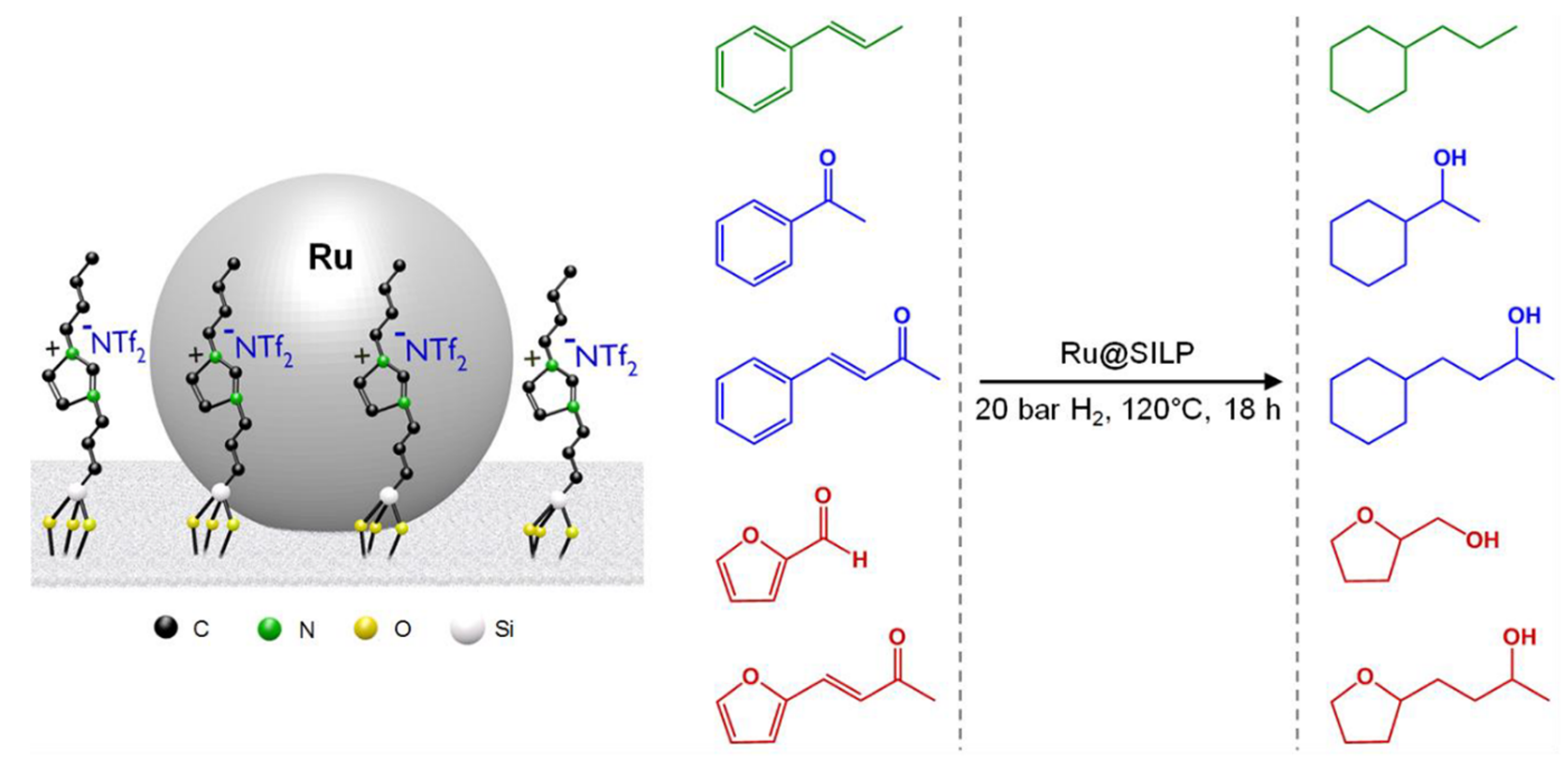
Hydrogenation of aromatic substrates containing various unsaturated functional groups using a Ru@SILP catalyst.

As part of our initial studies in this area, we observed that small Ru NPs generated by the hydrogenation of [Ru(2-methylallyl)2(cod)] or [Ru(cod)(cot)] on an imidazolium-based SILP are able to completely hydrogenate various aromatic substrates, including benzylideneacetone (Figure 6).^31^ In this case, the IL grafted on silica does not take part directly in the catalysis but contributes to the stabilization of the Ru NPs. Interestingly, biomass-derived furfuralacetone (last substrate shown in Figure 6) could be effectively converted to the corresponding saturated alcohol, which is a key intermediate in the production of various fuels and fuel additives from biomass (e.g., butyltetrahydrofuran, 1-octanol, dioctylether).^23^

**Figure 7:**
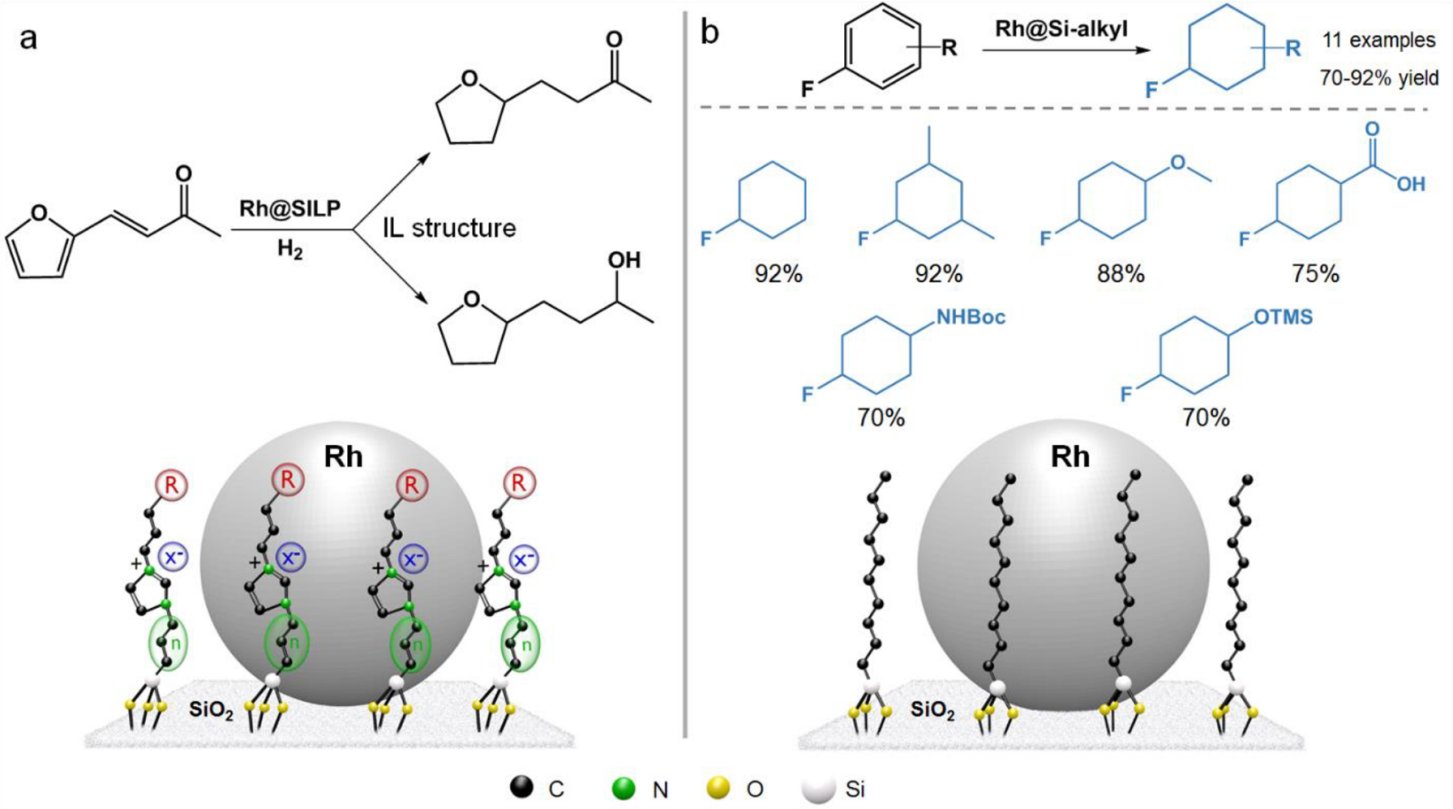
Rh@MMS as catalysts for the selective hydrogenation of (a) furfuralacetone; (b) fluoroarenes.

While solvents and ligands are known to control the formation of metal NPs in solution,^33^ the influence of chemisorbed molecular modifiers on the growth of NPs is far less studied. To investigate the influence of the structure of the molecular modifiers on the synthesis of NPs, we synthesized Rh NPs on a range of imidazolium-based SILPs with systematic variations of the IL structure (anion, *N*-alkyl chain length, spacer length; Figure 7a).^1^ While small (0.5–2 nm) and well dispersed Rh NPs were observed in all cases on the SILPs, the structure of the grafted IL was found to have a significant influence on the size of the NPs and on their activity for the full hydrogenation of furfuralacetone. At similar NP sizes (1.2 nm), Rh@SILP catalysts were found to be more active and much more stable under continuous flow conditions than a reference Rh@SiO_2_ catalyst. The characterization of Rh@SILP materials after catalysis (6 h on stream) did not evidence any leaching of the molecular modifier or of the metal or any growth of the metal nanoparticles. In sharp contrast, Rh NPs on Rh@SiO_2_ were found to aggregate, growing up to twice their original size over the course of the reaction. Interestingly, a decrease in the size of the spacer led to the formation of bigger NPs (2.0 nm) that were still highly active for C=C and furan ring hydrogenation but left the ketone intact.

The modification of the surface polarity by alkylsilanes was found to be highly beneficial for the use of Rh nanoparticles in the challenging selective hydrogenation of fluoroaromatics to fluorocyclohexanes (Figure 7b).^34^ The increase of the alkyl chains grafted on silica renders the environment of the Rh NPs apolar and hydrophobic, thus favoring the hydrogenation pathway over hydrodefluorination. Excellent yields of fluorinated cycloalkanes could be achieved for a broad range of substrates, matching or even surpassing that of molecular Rh-complexes as precatalysts and showing in addition the superior catalyst stability and reusability.^35^

Along the same line, Schüth and co-workers tuned the polarity of Ni_2_P@SiO_2_ materials through the chemisorption of organosilanes and used these catalysts for the hydrodeoxygenation of guaiacol. Fast catalyst deactivation due to the formation of water could be prevented by generating a nonpolar catalyst surface through the chemisorption of methyltriethoxysilane.^36^

Dupont and co-workers showed that the activity of imidazolium-based Pd@SILP catalysts for the partial hydrogenation of 1,3-cyclohexadiene can be modulated by changing the anion of the SILP, resulting in higher turnover frequencies for hydrophobic anions (PF_6_, NTf_2_; TOF = ca. 3 s^–1^) than for hydrophilic ones (Cl, NO_3_; TOF = ca. 1 s^–1^).^37^ The same group investigated the potential of confinement effects in catalytic systems composed of Au NPs immobilized on Al_2_O_3_-based SILPs in the hydrogenation of *trans*-cinnamaldehyde. From kinetic studies and the determination of kinetic isotopic effects, the authors concluded that the dynamic chemisorbed IL layers act as nanocontainers for the Au NPs and play a key role in controlling the mode of activation of H_2_ (homolytic vs heterolytic).^38^

Rossi and co-workers synthesized Pd NPs on silica surfaces modified with primary amine-functionalized ligands (propylamine, propylethylenediamine, propyldiethylenetriamine). In the presence of excess amine ligands, the Pd NPs were more stable and selective for the semihydrogenation of alkynes to alkenes than on bare SiO_2_, while the production of alkane was suppressed.^39^ Tao et al. prepared Pd NPs immobilized on IL-modified sepiolite and showed that the resulting catalyst was highly active and stable for the hydrogenation of alkenes. The presence of the IL functionalization was revealed to be important to allow the synthesis of small and well dispersed Pd NPs on the support. As a result, the IL-modified catalyst was found to be more active and more stable than classical Pd/C catalysts.^40^

These selected studies demonstrate that the molecular functionality incorporated on the support plays a crucial role in controlling the growth of the NPs and enhancing their stability. In addition, even if they are not reacting directly with the substrates, the molecular modifiers can significantly influence the reactivity observed, for example, through stereoelectronic interactions with the NPs, modification of the polarity and hydrophilicity of their environment, or confinement effects.

#### Bimetallic Monofunctional NPs@MMS Catalysts

**Figure 8:**
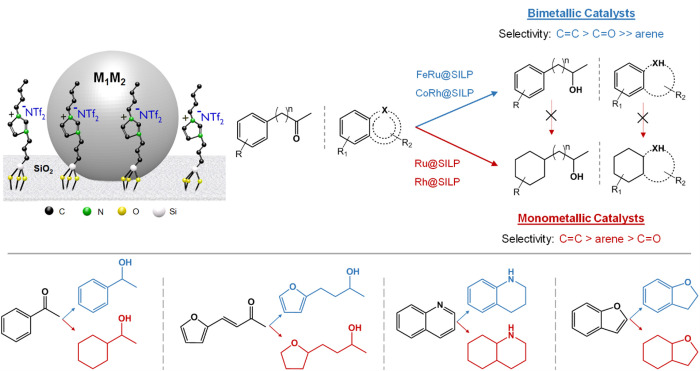
Selective hydrogenation of aromatic ketones and bicyclic heteroaromatics using bimetallic NPs@MMS catalysts.

As seen from the previous part, Rh, Ru, and Pd@MMS are excellent catalysts for the hydrogenation of olefins and arenes. However, the selective hydrogenation of C=O bonds in aromatic substrates for the production of aromatic alcohols is particularly difficult with these metals, which typically result in full hydrogenation. In fact, in many cases, the hydrogenation of aromatics occurs either in parallel or even faster than that of C=O units, for example, with heterogeneous Ru and Rh catalysts. The development of catalytic systems able to efficiently hydrogenate C=O bonds while leaving aromatics untouched is synthetically highly desirable, yet challenging. Following the general concept of Figure 6, it requires a polarized activation of dihydrogen while electronic and/or structural properties prevent the hydrogen transfer to the arenes. We have shown that bimetallic NPs immobilized on imidazolium-based SILPs are very promising candidates to combine these features (Figure 8).^2,31^ In particular, we synthesized a series of alloy-type Fe_*x*_Ru_100–*x*_@SILP catalysts using the complexes {Fe[N(Si(CH_3_)_3_)_2_]_2_}_2_ and [Ru(cod)(cot)] as precursors for the organometallic approach.^31^ The selection of organometallic precursors of a similar stability is important to ensure their simultaneous decomposition under the conditions applied and thus avoid the formation of monometallic NPs and/or core–shell structures. In addition, the presence of the IL layer chemisorbed on the support was found to be beneficial for the formation and stabilization of small and uniformly dispersed bimetallic Fe_*x*_Ru_100–*x*_ NPs on the SILP, which was not possible on unmodified SiO_2_ using the same conditions. While the monometallic Ru@SILP catalyst led to the full hydrogenation of the substrates, the bimetallic Fe_25_Ru_75_@SILP catalyst was found to be highly active and selective for the partial hydrogenation of aromatic ketones to aromatic alcohols. Intriguingly, we observed that not only was the arene hydrogenation suppressed almost completely but also, at the same time, the rate of C=O hydrogenation was ca. 4 times higher when using Fe_25_Ru_75_@SILP instead of Ru@SILP. This enhancement of the C=O hydrogenation rate can be explained by a more polarized activation of hydrogen on the FeRu surface than on the pure Ru surface as well as by an activation of the C=O bond on the more oxophilic FeRu surface. One possible reason for the lack of ring hydrogenation could be the disruption of the necessary arrangements of Ru atoms on the particle surface by the incorporation of Fe atoms. Dupont and co-workers used bimetallic FeRu NPs in IL media for the hydrogenation of CO_2_ to formic acid and heavy hydrocarbons. The CO_2_ hydrogenation pathway was dictated by both the basicity and hydrophilicity of the imidazolium IL and by the composition of the NPs. In particular, the use of bimetallic FeRu NPs resulted in enhanced selectivity toward the formation of heavy hydrocarbons as compared to monometallic Fe and Ru catalysts, presumably due to synergistic effects arising from the combination of a reverse water gas shift-active metal (Fe) and a Fischer–Tropsch-active metal (Ru (+Fe)).^41^

Recently, we prepared Co_*x*_Rh_100–*x*_@SILP catalysts with various Co/Rh ratios using [Co(cod)(cyclooctadienyl)] and [Rh(allyl)_3_] and applied them for the hydrogenation of aromatic ketones and bicyclic heteroaromatics (Figure 8).^2^ A very sharp switch in selectivity was observed between the Co_30_/Rh_70_ and Co_25_/Rh_75_ ratios. With a Rh content between 20% and 70%, the Co_*x*_Rh_100–*x*_@SILP catalysts possessed high activity and selectivity for the hydrogenation of C=O, C=C, and heteroaromatics but not for 6-membered aromatic rings. Above 75% Rh, the catalysts led to the full hydrogenation of all the substrates considered. In addition, synergistic effects arising from the alloying of Rh and Co were observed, and the Co_20_Rh_80_@SILP catalyst provided the highest C=O hydrogenation rate.

Zhao and co-workers recently showed that the selectivity of furfural hydrogenation using supported Pd_*x*_Ag_100–*x*_ nanoparticles as catalysts depends on the Pd/Ag ratio with the production of the fully saturated product for low Ag content (<20%) and the partially saturated product furfuryl alcohol at high Ag content (>50%).^42^ DFT calculations indicate that the adsorption free energy of the intermediate furfuryl alcohol on the Pd(111) surface is inversely proportional to the Ag content in PdAg bimetallic NPs. Rana and Jonnalagadda showed that bimetallic Ni_50_Cu_50_ NPs supported on organo-functionalized graphene oxide allow the selective hydrogenation of nitrophenol and cinnamaldehyde to aminophenol and cinnamyl alcohol, respectively. The organic amine groups chemisorbed on graphene oxide play the role of stabilizers, increasing the binding capacity of the NPs.^43^ Han and co-workers prepared bimetallic Cu_*x*_Ru_100–*x*_ NPs on bentonite functionalized with a guanidinium IL. The IL was found to be crucial for the formation of the NPs and their stabilization under reaction conditions (190–240 °C, 25–100 bar H_2_, 18 h). In addition, bimetallic catalysts were more active and selective for the hydrogenolysis of glycerol to 1,2-propanediol than the monometallic Cu and Ru versions with particularly good results obtained for the Cu_25_Ru_75_ catalyst (100% conversion, 87% selectivity for 1- and 2-propanol).^21^

These examples demonstrate that the selectivity of the hydrogenation reactions, and especially the catalyst’s ability to hydrogenate polarized C=O units versus aromatic rings, can be effectively and finely controlled through the preparation of bimetallic NPs with tunable metal ratios on SILP-type materials. Intriguingly, the incorporation of 3d metals in nanoalloys does not modify the performance of noble metals only via “dilution” effects but allows the generation of very significant synergistic effects leading to the acceleration of some transformations while others are completely shut down. The modularity of the organometallic approach opens the possibility to explore almost any combination of metals by systematic variation of the composition, providing a unique platform for optimization and mechanistic studies.

#### Monometallic Multifunctional NPs@MMS Catalysts

This third part will cover monometallic nanoparticles immobilized on MMSs where the molecular modifier possesses a functionality that is directly involved in the catalysis. These materials fall in the class of multifunctional catalysts, which currently attract considerable attention not only for the production of fine chemicals and pharmaceuticals but also for the conversion of oxygen-containing renewable feedstocks and platform chemicals into value-added chemicals and fuel components.^44,45^ We particularly used this approach to develop catalytic systems able to perform selective hydrodeoxygenation reactions. As described in Figure 5, hydrodeoxygenation reactions are generally facilitated by the presence of H^+^, and Brønsted acid functionalities can be introduced in the molecular modifiers in many different ways. Lewis acidity can also be provided through the modifier, whereby the anions of IL-type structures offer a versatile opportunity to fine-tune the acid strength.

**Figure 9:**
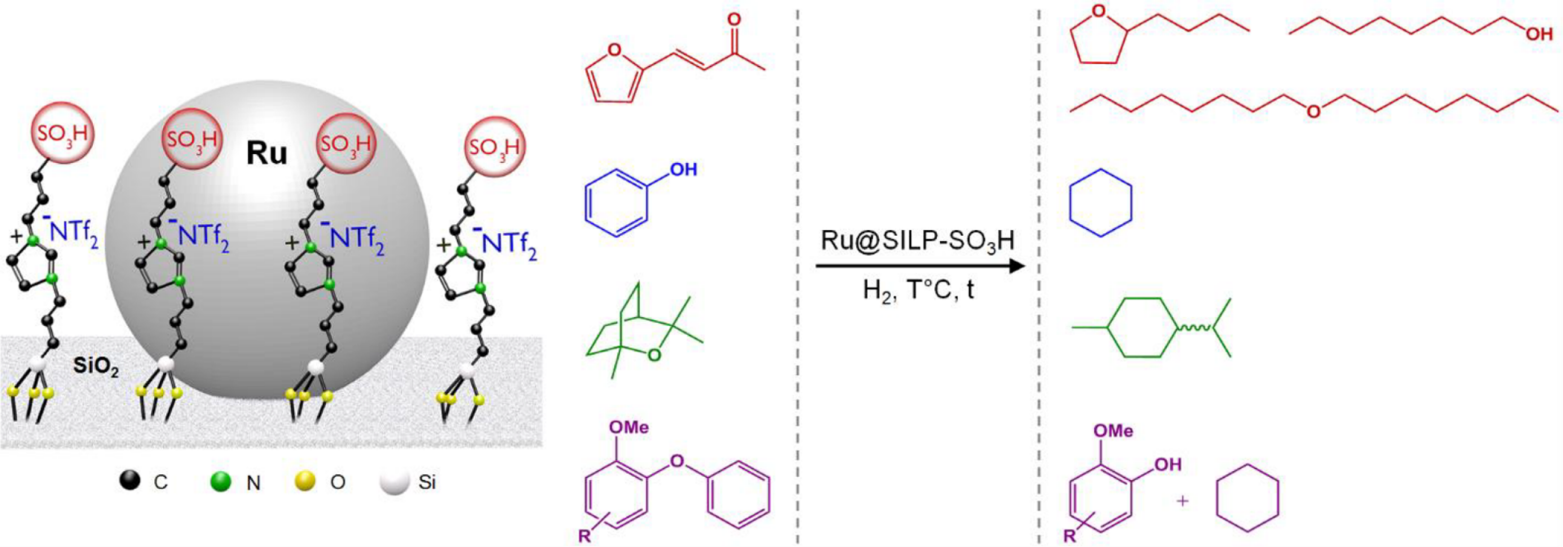
Hydrodeoxygenation of various substrates using a Ru@SILP-SO_3_H catalyst.

Our group has shown that bifunctional catalysts made of Ru NPs embedded in sulfonic acid functionalized imidazolium-based SILPs (Ru@SILP-SO_3_H) are excellent hydrodeoxygenation catalysts (Figure 9).^23,46−48^ In this case, the hydrogenation steps are performed by the Ru NPs, while the −SO_3_H groups of the support are responsible for the deoxygenation activity. This reactivity was exemplified for the full hydrodeoxygenation of phenol,^46^ eucalyptol,^47^ and furfuralacetone.^23^ In the latter case, it was demonstrated that tuning the amount of −SO_3_H functions grafted on the support allowed the control of the acidity of the material and hence the selectivity of the reaction, providing individual access to butyltetrahydrofuran, octanol, or dioctylether, which are promising fuels and fuel additives. The combination of the hydrogenation (Ru NPs) and deoxygenation (−SO_3_H) active sites on a single-support was confirmed to generate synergistic effects, leading to enhanced hydrodeoxygenation activities of Ru@SILP-SO_3_H as compared to a mixture of its single components.^46^ Recently, we further illustrated the versatility of Ru@SILP-SO_3_H through its application in selective hydrogenolysis of various substituted diaryl ethers.^48^ The presence of the strong acid sites in combination with the Ru NPs was found to be the key to high ether bond cleavage activity. In several of these reactions, the Ru@SILP-SO_3_H material was used for several hours under continuous flow conditions without leaching of the active phase or growth of the Ru NPs, highlighting its stability.^46−48^

Kim and co-workers used Pd NPs supported on a Zr-based MOF deposited on sulfonated graphene oxide (Pd/UiO-66@SGO) as a bifunctional catalyst to convert monosaccharides into 2,5-dimethylfuran in one pot. In this case, the Brønsted acidic functionalities (−COOH, −OH, and – SO_3_H groups of the SGO support) are responsible for the deoxygenation of fructose to 5-hydroxymethylfurfural, while the Pd NPs perform the hydrogenolysis and hydrogenation of 5-hydroxymethylfurfural to 2,5-dimethylfuran. Following this one-pot strategy, excellent 2,5-dimethylfuran yields (up to 70.5% at 160 °C, 10 bar H_2_) were obtained.^49^ Hou and co-workers have shown that supporting Ru NPs on sulfonic acid-functionalized silica (Ru@SiO_2_–SO_3_H) produces a bifunctional catalyst possessing a high activity for the hydrogenolysis of cellulose into sorbitol. Using XPS and FT-IR with pyridine as the molecular probe, the authors evidenced strong electronic interactions between the sulfonic groups and the Ru NPs. The bifunctional catalyst was found to be more selective toward the formation of sorbitol than a physical mixture of its individual components (Ru@SiO_2_ + SiO_2_–SO_3_H) with, in particular, less side reactions of the sorbitol presumably due to the “poisoning” of the Ru NP surface by the sulfonic acid functionalities.^50^ Qu and co-workers prepared Ru NPs immobilized on benzenesulfonic acid-functionalized graphene. The resulting bifunctional catalyst was found to be highly active for the conversion of biomass-derived levulinic acid to γ-valerolactone (ca. 640 h^–1^ at 50 °C, 20 bar H_2_). In this case, the Ru NPs are responsible for the hydrogenation of the levulinic acid to 4-hydroxyvaleric acid while the sulfonic acid-functionalized surface catalyzes the dehydration of 4-hydroxyvaleric to γ-valerolactone.^51^

**Figure 10:**
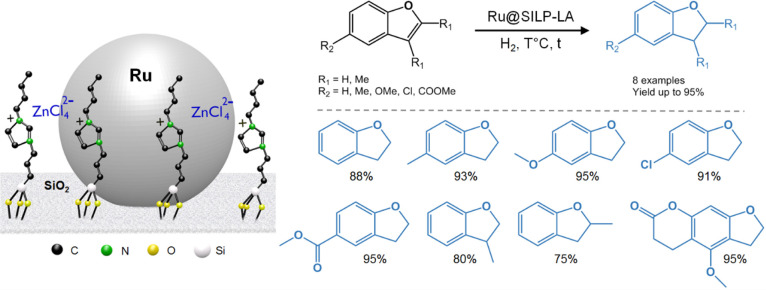
Selective hydrogenation of benzofuran derivatives using a Ru@SILP-LA catalyst.

Recently, we reported the preparation of Ru NPs on a Lewis acidic SILP containing chlorozincate anions (Ru@SILP-LA).^3^ In-depth characterization of the catalyst using XPS and electron microscopy techniques (STEM-HAADF-EDX) evidenced strong interactions between the chlorozincate species (mainly ZnCl_4_^2–^) and the Ru NPs surface. Ru@SILP-LA was found to be highly active, selective, and stable for the partial hydrogenation of benzofurans with catalytic properties superior to Ru/C and nonfunctionalized Ru@SILP catalysts (Figure 10). The obtained dihydrobenzofuran derivatives are key building blocks for the production of pharmaceuticals and bioactive molecules.^52^

**Figure 11:**
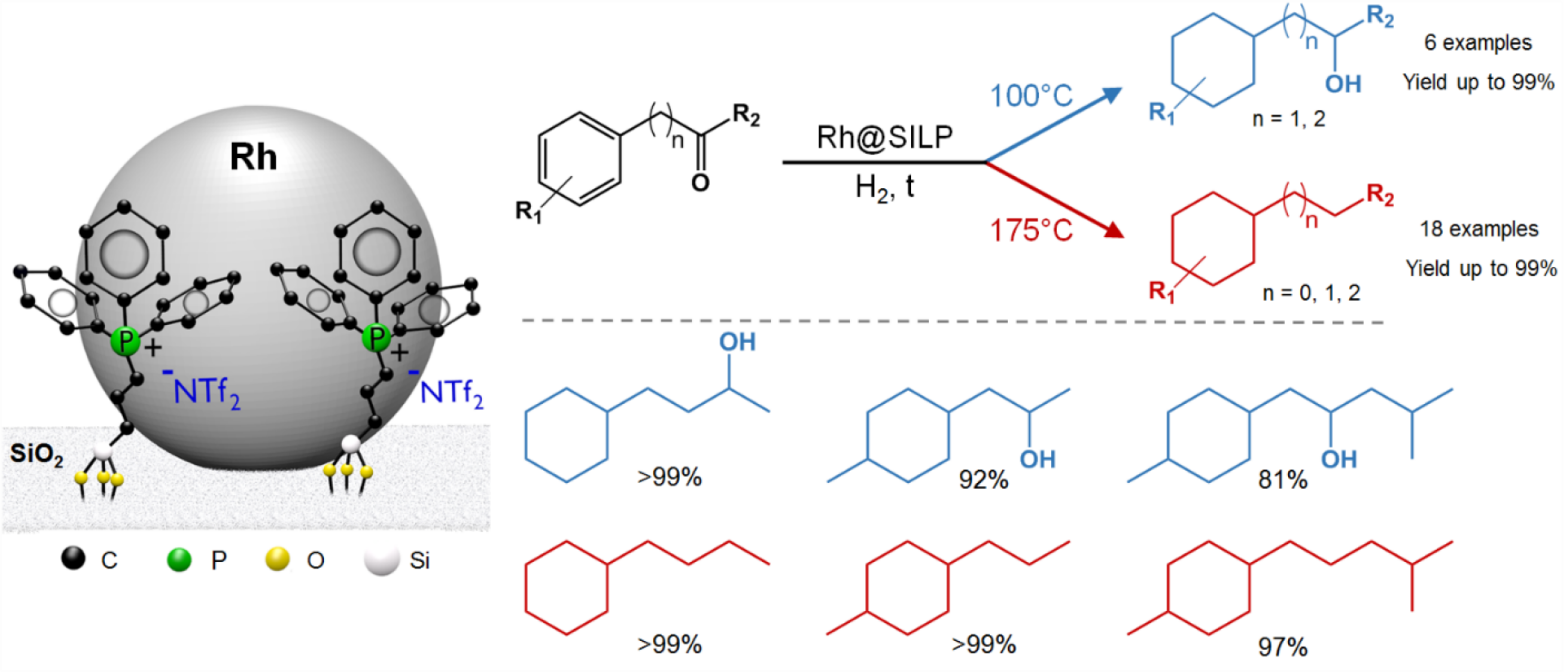
Temperature-controlled hydrogenation and hydrodeoxygenation of aromatic ketones using a phosphonium-based Rh@SILP catalyst.

Interestingly, a catalyst possessing a high activity for the hydrodeoxygenation of aromatic ketones was obtained without the addition of a strong acid when Rh NPs were produced on a phosphonium-based SILP comprising the NTf_2_ anion. The deoxygenation activity was attributed in this case to the formation of Lewis acidic Rh–F species due to the partial decomposition of the NTf_2_ anion on the NPs’ surface.^32^ The resulting balance between hydrogenation and hydrogenolysis activity enabled precise temperature control, and thus, the selectivity could be switched to produce aromatic products with either alcohol or alkyl side chains (Figure 11). The produced alkyl cyclohexanes are important kerosene-type fuels for the transportation sector,^53^ while the hydroxyl-containing cyclohexane derivatives are used as building blocks for the production of coating agents and pharmaceuticals.^54,55^

#### Multimetallic Multifunctional NPs@MMS Catalysts

We have seen in the previous parts the benefits associated with the use of bimetallic NPs@MMS in hydrogenation reactions and to NPs@MMS possessing reactive functionalities for the cleavage of C–O bonds by hydrogenolysis. Consequently, the combination of these features appears particularly attractive to access catalytic systems with tailor-made reactivity.^56,57^ To highlight this potential, we recently reported the preparation of a bimetallic bifunctional catalyst composed of Fe_25_Ru_75_ NPs immobilized on a sulfonic acid-functionalized SILP.^4^ A major challenge in the preparation resulted from the incompatibility of the Fe precursor complex with the acid functionality on the support. This would apply to many 3d metal complexes, resulting in a significant limitation. To overcome this problem, we developed a method for postmodification of NP@SILP with additional molecular modifiers. The preparation of such catalytic systems involves first the synthesis of the bimetallic NPs on a SILP without acid sites followed by physisorption of sulfonic acid-functionalized imidazolium-based IL. Due to the strong electrostatic interaction between the chemisorbed and physisorbed IL-type structures, close contact between the active components is assured, similar to what is known from SCILL catalysts in classical heterogeneous catalysis.^15^

**Figure 12:**
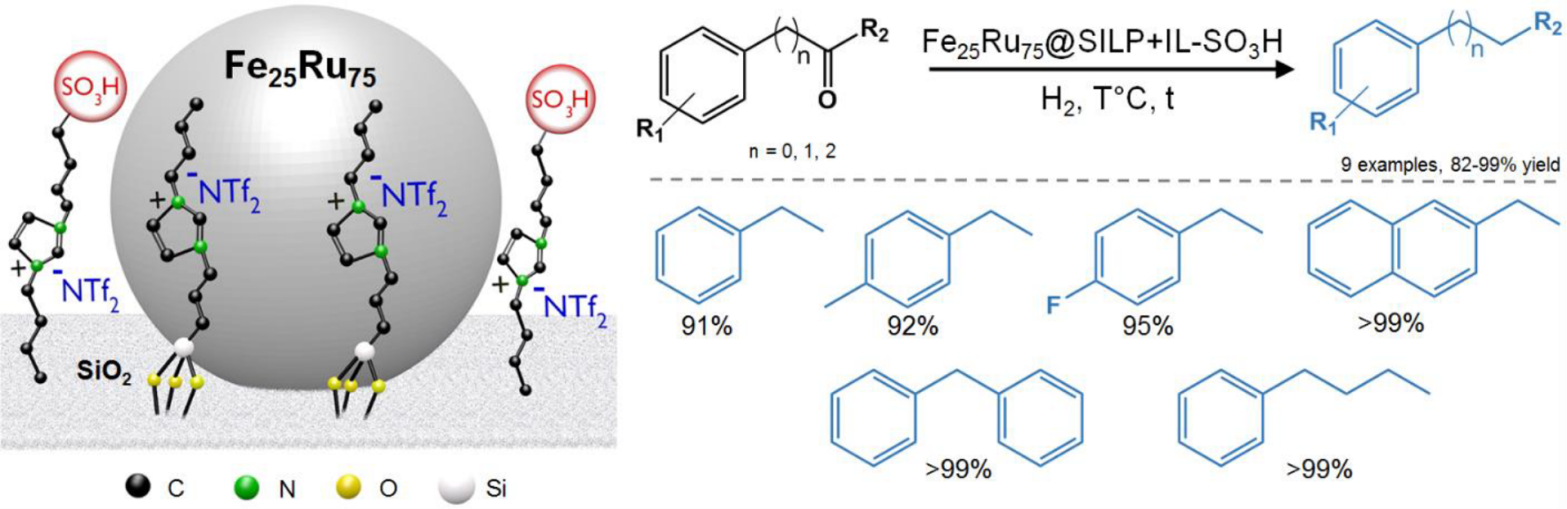
Selective hydrodeoxygenation of benzylic and nonbenzylic ketones using a Fe_25_Ru_75_@SILP+IL-SO_3_H catalyst.

The Fe_25_Ru_75_@SILP+IL-SO_3_H catalyst obtained by this method was found to be highly active, selective, and stable for the hydrodeoxygenation of various benzylic and nonbenzylic ketones to alkyl aromatic derivatives (Figure 12). In this example, the Fe_25_Ru_75_ NPs are responsible for the highly selective hydrogenation activity, while the presence of the −SO_3_H acid sites unlocks the deoxygenation of the resulting alcohol. The synergistic effects generated by the intimate contact between the NPs and the SO_3_H functionality allowed the reaction steps requiring heterolytic H_2_ activation to proceed smoothly while effectively shutting down aromatic hydrogenation. Remarkably, the hydrogenolysis is not restricted to benzylic positions but occurs with even higher rates for C–O bonds remote from the aromatic moiety. The catalyst system could be applied to a broad scope of substrates offering a green alternative to the classical Clemmensen and Wolff-Kischner reductions and opening the way to an efficient upgrade of biosourced aromatic ketones^58^ through hydrodeoxygenation.

**Figure 13:**
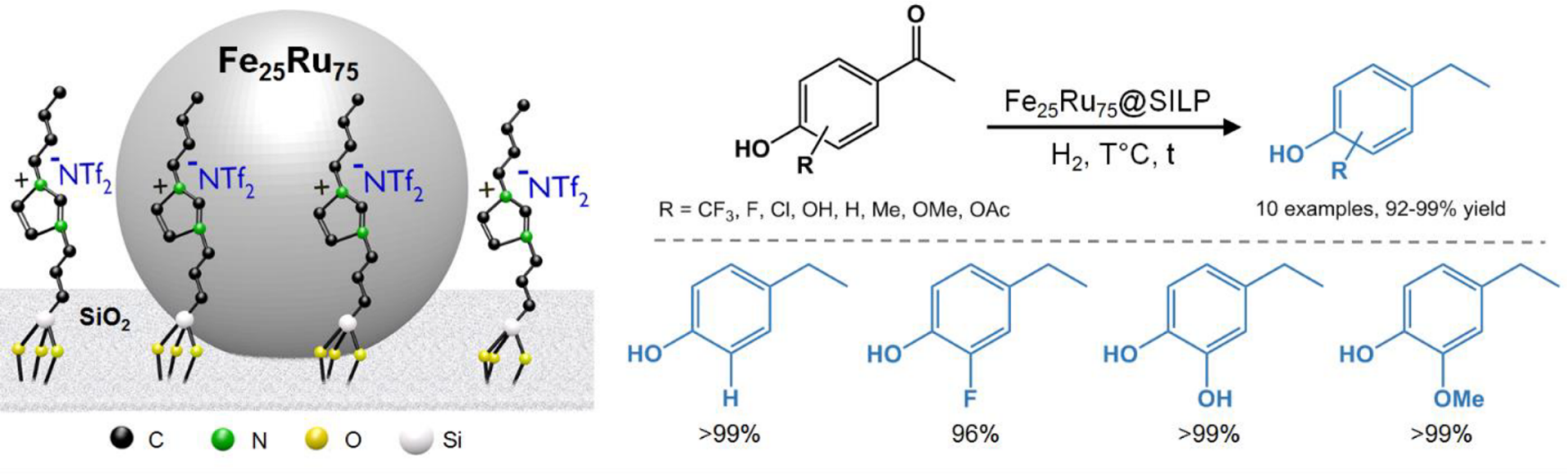
Selective hydrodeoxygenation of hydroxyacetophenone derivatives using a Fe_25_Ru_75_@SILP catalyst.

Interestingly, while Fe_25_Ru_75_@SILP+IL-SO_3_H could not selectively hydrodeoxygenate hydroxyacetophenone derivatives due to various acid-catalyzed side reactions, we recently showed that the bimetallic acid-free Fe_25_Ru_75_@SILP catalyst is very efficient and selective for this transformation (Figure 13).^59^ The mesomeric effects activating the ketone and stabilizing the intermediate carbocation were found to be important to observe high hydrodeoxygenation activity. This provides access to a broad range of ethylphenol derivatives that are important building blocks for the production of fine chemicals, polymers, and pharmaceuticals.^60,61^

## Conclusions and Perspectives

**Figure 14:**
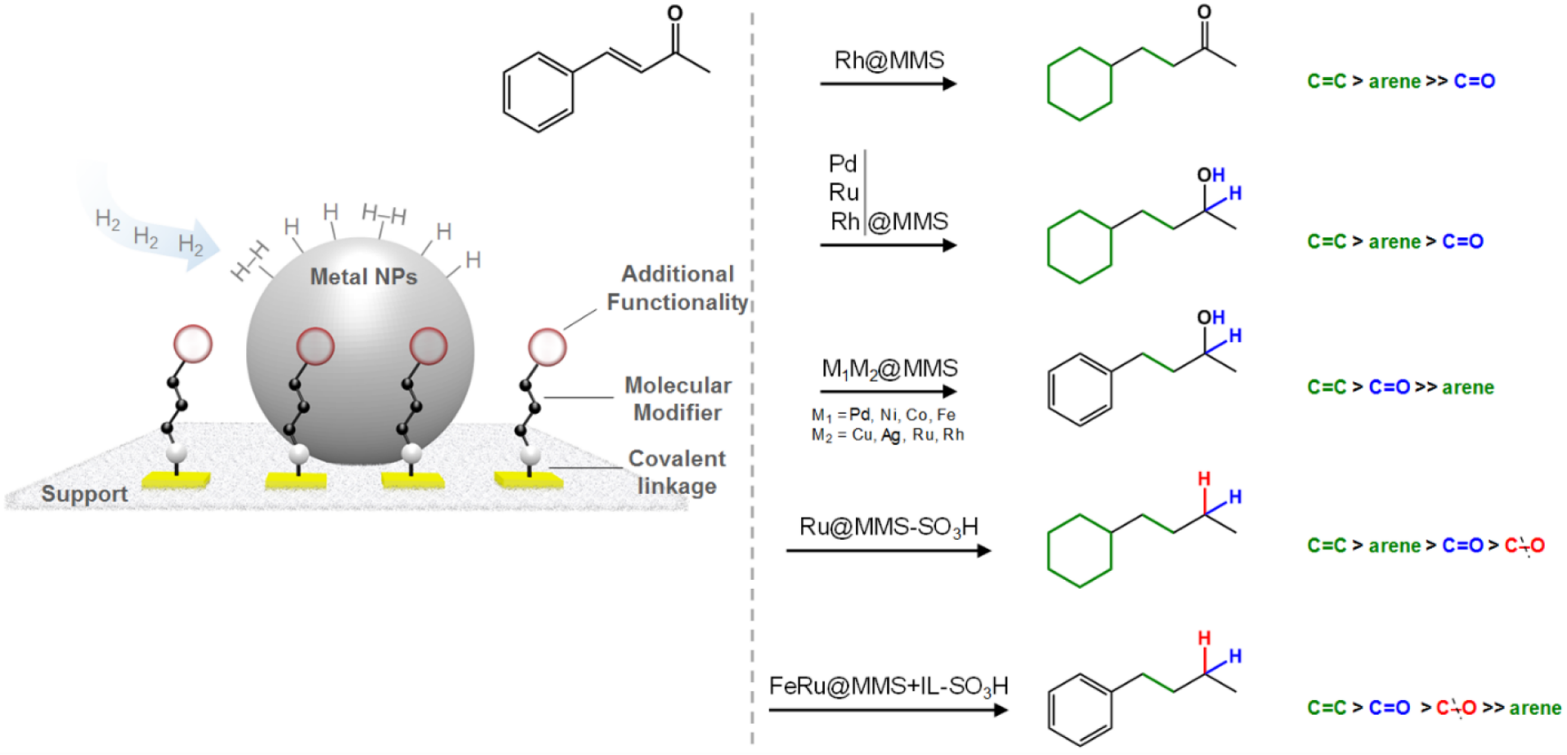
Controlled hydrogenation and hydrogenolysis of benzylideneacetone using NPs@MMS catalysts.

The organometallic approach to generate metal nanoparticles on molecularly modified surfaces, NPs@MMS, is emerging as a highly attractive strategy for the rational design and systematic preparation of excellent catalysts at the interface of molecular (“homogeneous”) and materials (“heterogeneous”) catalysis. The preparation methods are highly versatile, allowing the production of multimetal and multifunctional systems. In particular, this modularity allows one to fine-tune the balance between homolytic and heterolytic activation of H_2_ and, thus, the selectivity of hydrogenation and hydrogenolysis reactions. This can be showcased nicely using benzylideneacetone as the model substrate, where the modification of the NP composition and of the molecular modifier allowed one to selectively target each of the possible products by controlling the relative order of the hydrogen addition to the functional groups (Figure 14). In comparison with conventional NPs@support catalysts, NPs@MMS materials prepared by chemisorption were demonstrated to offer the potential benefits of enhanced catalytic properties (activity, selectivity) as well as superior stability and reusability. When grafted effectively, the molecular modifiers lead to very little and often even no leaching of the active phase or changes in the metal nanoparticle size and distribution during catalysis.

The synthetic potential of such controlled transformations can help to exploit the green chemistry principles for the existing chemical value chain as well as for novel biomass-derived product streams.^29,30,44,45,57,58^ Recently demonstrated examples that are very challenging to achieve with classical heterogeneous or homogeneous catalysts include the selective hydrogenation of bicyclic heteroaromatics, fluorinated aromatics, aryl ether cleavage, and hydrogenation and deoxygenation of side chains in aromatics. Most recently, it has been demonstrated that the introduction of reactive functional groups can open the path to adaptive catalytic systems enabling reversible control over their performance by applying external stimuli.^62^ The manifold opportunities arising from the emerging design criteria motivate future developments in this direction.

While the interest of NPs@MMS materials as multifunctional catalysts for synthesis applications is clear, significant efforts are still required to fully unlock their potential. Although the importance of intimate contact between the NPs and the modifier has been demonstrated in particular for the SILP-type supports, the fundamental understanding of the interactions between the different catalyst components and their implications in catalysis currently remains largely elusive. Obviously, this is of crucial importance for the rational development of NPs@MMS catalysts and should be investigated in detail using a combination of characterization and computational techniques. A particular challenge in this context is the need for *in operando* characterization under catalytic conditions to understand the structural and reactive dynamics beyond the static structures before and after catalysis. In addition, detailed kinetic studies and determination of kinetic isotopic effects will contribute to a better understanding of hydrogen activation processes in these complex catalytic systems.

The methodology toolbox and the selected examples presented in this Account evidence that NPs@MMSs are now widely accessible and that the knowledge-based design and preparation of NPs@MMS catalysts offer new pathways for challenging transformations. As the available information is assembled from individual examples to systematic classes of metal-functionality combinations, the capacity to develop catalysts with increasing complexity and controlled reactivity by this approach will benefit from interdisciplinary research efforts by organometallic chemists, catalysis experts, synthetic chemists, and process engineers. We therefore hope that this Account will encourage researchers to include NPs@MMSs in the portfolio of multifunctional catalytic systems.
